# What can family medicine providers learn about concussion non-disclosure from former collegiate athletes?

**DOI:** 10.1186/s12875-018-0818-2

**Published:** 2018-07-27

**Authors:** Elizabeth A. Beverly, Todd R. Fredricks, Andrew Leubitz, Benjamin R. Oldach, Daniel Kana, Michael D. Grant, Jonathon Whipps, Emily H. Guseman

**Affiliations:** 1 Department of Family Medicine, Ohio University Heritage College of Osteopathic Medicine, Athens, OH 45701 USA; 2 The Diabetes Institute, Ohio University, Athens, OH 45701 USA; 3 Department of Psychology, Ohio University, Athens, OH 45701 USA; 4 The Graduate College, Ohio University, Athens, OH 45701 USA

**Keywords:** Concussions, Concussion non-disclosure, Chronic traumatic Encephelopathy, Sports, Qualitative methods, Family medicine

## Abstract

**Background:**

Despite the risks, concussion symptoms often go underreported by athletes, leading to delayed or forgone treatment and increased potential for concussion recurrence. One of the most serious long-term consequences of sports-related concussions is Chronic Traumatic Encephelopathy (CTE), a disorder associated with progressive neurological deterioration. The purpose of this study was to explore former collegiate athletes’ understanding of concussions and motivations behind concussion non-disclosure in order to better assist family medicine providers in screening for and managing a history of concussions.

**Methods:**

Informed by the theoretical framework Social Cognitive Theory, we conducted focus groups with former collegiate athletes using a field-tested discussion guide. Discussions were transcribed, coded, and analyzed via content and thematic analyses using NVivo 10 software.

**Results:**

Thirty-two former collegiate athletes (24.5 ± 2.9 years old, 59.4% female, 87.5% white) participated in 7 focus groups. Three predominant themes emerged: 1) Concussions are Part of the Game: Participants believed that concussions were part of sports, and that by agreeing to play a sport they were accepting the inherent risk of concussions. Importantly, many were not familiar with concussion symptoms and what constituted a concussion; 2) Hiding Concussion Symptoms: Participants said they often hid concussion symptoms from coaches and trainers in order to avoid being taken out of or missing games. Participants were able to hide their concussions because most symptoms were indiscernible to others; and 3) Misconceptions about Concussions in Low Contact Sports: Several participants did not understand that concussions could occur in all sports including low contact or noncontact sports. The former athletes who participated in low contact sports and experienced concussions attributed their concussions to personal clumsiness rather than their sport.

**Conclusions:**

Family medicine providers as well as coaches, athletic trainers, teachers, and parents/guardians should reinforce the message that concussions can occur in all sports and inform patients about the signs and symptoms of concussions. Further, providers should ask all patients if they engaged in high school or collegiate athletics; and if yes, to describe their hardest hit to their head in order to obtain a complete medical history.

## Background

Head injuries are prevalent in collision and high contact sports such as soccer, rugby, American football, mixed martial arts, boxing, judo, and karate as well as winter sports such as ice hockey, skiing, ice skating, and snowboarding [1]. Popular sports differ by country and therefore the sport associated with the highest number of head injuries often varies by country. For example, in Japan, judo is the leading cause of catastrophic head injury [1] whereas, in New Zealand, the leading cause of head injury was rugby [2]. In Australia, Australian Rules football, rugby league, and rugby union have the highest rates of heady injury compared to any other team sport in the world, with 3 to 10 concussions per 1000 playing hours [3–6]. Worldwide, the most common sports-related head injury is a concussion or mild traumatic brain injury (mTBI) [1]. The most serious sequela of sports-related concussions include acute subdural hematoma, acute epidural hematoma, cerebral contusion, traumatic cerebrovascular accidents, diffuse brain swelling, diffuse axonal injury, skull fractures, Chronic Traumatic Encephelopathy (CTE) [1]. Chronic Traumatic Encephelopathy (CTE), a progressive disorder associated with repetitive head trauma [7–9], has received a great deal of attention from researchers and the media in the last several years.

In the United States (US), approximately 3.8 million sports-related concussions occur annually accounting for nearly 10% of all sports-related injuries [10–12]; whereas, approximately 300,000 sports-related head injuries occur in Europe per year [13]. Thirty percent of all sports-related concussions result in emergency department visits [14–16]. Of special interest is neuropathology among former players of American football, particularly those who played at the collegiate and professional levels. Recent reports from a convenience sample of deceased American football players indicates that CTE may be present in as many as 87% of former football players [17] and that age of first football exposure predicts neuropsychiatric impairment in adulthood [18]. Behavioral and mood symptoms among both mild and severe cases of CTE include impulsivity, depression, apathy, anxiety, hopelessness, explosivity, verbal and physical violence, suicidality, substance use, and post-traumatic stress disorder [17, 18]. Cognitive symptoms consist of memory loss, executive dysfunction, attention deficits, language and visuospatial problems, gait instability, and slowness of movement [17, 18]. Findings from a convenience sample of deceased American football players with CTE showed that the most common cause of primary death was neurodengerative [17]. Thus, the high rate of behavioral, mood, and cognitive symptoms as well as neurodegenerative disease observed in cases of CTE are of grave concern for athletes at risk for repetitive head trauma.

Despite the risks, concussion symptoms often go underreported by athletes, leading to delayed or forgone treatment and increased potential for concussion recurrence [19–21]. Signs and symptoms of concussions include but are not limited to: nausea, vomiting, headache, visual problems, loss of consciousness, cognitive impairments (e.g., difficulty concentrating, forgetfulness, confusion), sleep disturbances, and emotional changes (e.g., irritability, sadness, nervousness) [10, 22]. Although most concussion symptoms resolve within a week, symptoms may persist for months to years for some athletes [23, 24]. In recent years, symptom underreporting has driven the emergence of novel testing to develop an objective measure of concussive symptoms; however, symptom-based reporting still remains a key component of concussion evaluation among athletes [10]. Even if symptoms of a concussion are correctly identified and diagnosed, however, there is no consistent evidence-based precedent to determine when an athlete can safely return to play [25]. Despite increased awareness about symptom underreporting and the role symptom reporting plays in concussion evaluation, research that explores athletes’ decision-making process to report or not report symptoms is limited.

Exploring athletes’ understanding of concussions may be useful information for family medicine providers overseeing the primary care of children, adolescents, and young adults participating in sports. Further, understanding the motivations behind concussion non-disclosure may help family medicine providers communicate with current athletes by helping them navigate conversations via a specific line of questioning during a medical visit. Moreover, former athletes will continue to receive primary care from family medicine providers and providers will need to know their concussion history in order to evaluate the presence, severity, and prognosis of their concussion(s) in order to inform future management. Finally, providers will need to monitor their patients through the days, weeks, months, and years after a head injury and assess the degree of emotional difficulties as well as cognitive, memory, and executive functioning deficits over time. Therefore, the purpose of this qualitative study was to explore former collegiate athletes’ understanding of concussions and motivations behind concussion non-disclosure. Our research question was: Why do athletes hide concussions from teammates, coaches, and trainers?

## Methods

### Research

This qualitative focus group study explored participants’ understanding of concussions and motivations behind concussion non-disclosure among graduate and osteopathic medical students who formerly participated in undergraduate collegiate athletics. Focus groups are a qualitative technique in which data are collected through a semi-structured group interview of approximately five to twelve individuals [26]. The premise of focus group research is that attitudes, beliefs, perceptions, and expectations are not developed in isolation but rather through interaction with others [27, 28]. Thus, questions are asked in an interactive group setting where participants are free to talk with other group members. In this study, focus groups were selected to gain insight into athletes’ experiences with concussions and concussion non-disclosure in a convenient sample of former collegiate athletes. We followed the consolidated criteria for reporting qualitative research (COREQ) [29].

### Sample

Purposive sampling strategies [26] were employed to recruit English-speaking adults who played a collegiate sport (e.g., football, soccer, basketball, lacrosse, field hockey, baseball, softball, wrestling) at the Division I, II, or III level (Note: three participants competed on traveling club teams, in which they had to tryout in order to make the team), and were currently enrolled in a graduate or medical degree program at the University. In the US, collegiate sports are classified by divisions, such that Division I represents the highest level of athletic competition at the largest universities with partial to full athletic scholarships, Division II represents mid-level competition at mid-size universities with fewer financial resources compared to Division I school, and Division III represents lower-level competition at the smallest colleges; Divisions III schools are not allowed to offer athletic scholarships. Based on prior concussion reporting research [30–32], former collegiate athletes were selected to describe their experiences without pressure or fear of repercussions from coaches, trainers, and teammates for participating in the study. Prior diagnosis of a concussion was not required for participation as we were interested in former athletes’ understanding of concussions and concussion non-disclosure. Participants were recruited via the University graduate and medical student email listservs containing approximately 5000 students. In the US, approximately 7% of male and female high school students transition to participate in collegiate athletics. Thus, of the 5000 University graduate and medical students, we estimated that 350 students participated in college sports. Potential participants were contacted via email by University owned and matained listservs distributed by the Principal Investigator (EAB) and word of mouth. The research team distributed the recruitment email on October 16, 2013; a reminder email was sent one week later. Students responded to the email to express an interest in participation. All students who expressed an interest to participate were included in the study.

The University Institutional Review Board approved the study protocol IRB number 13X181. All participants provided written informed consent prior to participation and received a $15.00 gift card as compensation for their time. Participants were explicitly informed that there were no “right” or “wrong” responses to the focus group questions, and that none of the information they provided would be used to evaluate their academic performance at the University or medical school in any way. Further, participants were told they could decline to participate at any time in the study process without explanation.

### Theoretical framework

The semi-structured focus group discussion guide was based on Bandura’s Social Cognitive Theory (SCT) [33]. Implementing a theoretical framework was essential to understanding factors that influence concussion non-disclosure. SCT may help describe how environmental influences affect an individual’s reporting decisions about a concussion [33]. SCT represents the dynamic interaction of individual-level behavioral, personal, and environmental factors as demonstrated through a triadic causation reciprocal model [33]. Specifically, an athlete’s environment may influence concussion reporting behavior by influencing how he/she perceives what will happen after reporting a concussion. As outlined by the tenets of SCT, the environment directly influences the athlete’s behavior of reporting symptoms. For example, an athlete may witness a teammate reporting a concussion and receiving backlash from coaches and other teammates, therefore, influencing the likelihood of that athlete reporting a concussion. Today’s media adds another dimension to the athlete’s environment. Not only does the athlete have his/her own experience and teammates’ experiences with concussions, but also their attitude and behavior may be influenced by high profile athletes’ actions [34]. Thus, environmental factors may contribute to the stigma surrounding concussions and concussion reporting.

### Data collection

The research team devised a semi-structured discussion guide using the SCT framework and field-tested it for flow and clarity of the questions with a group of five participants (see Table 1). Once the discussion guide was finalized, data collection commenced. Focus groups were segmented by gender to account for potential gender differences in concussion non-disclosure. Specifically, we separated female participants from male participants by focus groups to remove any undue influence in the discussion of sensitive information about the understanding of concussions and motivations behind concussion non-disclosure. This segmentation plan allowed researchers to use shared experiences as a springboard for deeper examination and discussion of related issues, thus promoting greater depth in understanding without the potential influence or constraint exerted by members of the opposite gender. Focus groups continued until all interested students had participated.

**Table 1:** Focus group discussion guide for study participants

1.	What is a concussion?
	Probe. What are the signs and symptoms of a concussion?
2.	Have you ever experienced a concussion? Please describe.
	Probe. Did a trainer/coach or physician diagnose you with a concussion?
	Probe. Did you diagnose yourself?
	Probe. What symptoms did you experience?
	Probe. Did you miss any practice time or games?
3.	Have you ever hit heads with an opponent or teammate while playing your sport? Please describe.
	Probe. How did you feel after it happened? How did you feel the next day?
	Probe. Did you miss any practice time or games?
	Probe. Did you think this could have been a concussion? Why or why not?
4.	If you have ever had a concussion, did you tell anyone at the time that it happened or did you hide it?
	Probe. Why did you hide it? Please explain
5.	Did any of your teammates every have a concussion and hide it from a coach or trainer?
	Probe: Why do you think they hid it? Please explain.
6.	Did you ever play your sport when you should not have because of a concussion or head injury? Please explain.
7.	What did the coach expect from you as a player if you had a concussion or head injury (diagnosed concussion or not diagnosed concussion?)
8.	How did you feel if a teammate did not play in a game because of a concussion or head injury?
9.	Did a coach/trainer ever tell you to play even if you had a concussion or head injury?
10.	Did a parent ever tell you to play even if you had a concussion or head injury?
11.	Did fear of getting a concussion affect how you played the game?
12.	Is there a stigma in your sport about concussions? If so, what is it?
	Probe. Do you think this differs by gender?
13.	What advice would you give current athletes playing your sport about concussions?
14.	What advice would you give coaches, physicians, and parents about concussions?

Focus groups were conducted at University sites (conference rooms and study rooms). Trained moderators asked participants broad, open-ended questions about their understanding of concussions, personal and teammates’ experiences with concussions, and concussion non-disclosure. A female moderator, an Assistant Profession in Family Medicine (EAB), conducted the women’s focus groups and male moderators, three medical students and one Lecturer in Psychology (BRO, AL, DK, MDG), took turns conducting the men’s groups. All moderators were trained by an expert in qualitative methods (EAB). When the medical students and Lecturer in Psychology (BRO, AL, DK, MDG) were not serving as the moderator, they served as co-moderators for the focus groups by writing notes to capture key points (i.e., written accounts of what happened during focus groups) as well as observations (e.g., participant nonverbal communication, expressions, behaviors) about the discussions. At the end of each focus group, moderators and co-moderators met to share impressions and observations. All focus group discussions were audio-recorded and transcribed verbatim; participants’ names and identifiers were removed to protect their confidentiality. Participants also completed a brief demographic form and history of head injury questionnaire. Importantly, on the demographic form, participants were asked to name the sport they participated in during college. Sports were classified as collision sports or sports in which players collide as part of the game (e.g., rugby, American football), high contact sports or sports which require physical contact between players (e.g., soccer, lacrosse, basketball, water polo), and low contact sports or sports that have minimal or no means of touching other players (e.g., track and field, swimming).

### Data analysis

The multidisciplinary research team, consisting of a health behaviorist/qualitative methodologist (EAB), three medical students (AL, BRO, DK), and a health psychologist (MDG), analyzed data using content and thematic analyses [27]. The research team included two professors (EAB, MDG) who teach at the university where the participants attended. In addition, the three medical students (AL, BRO, DK) were current students at the medical school and peers of some of the participants. Specifically, the research team summarized the discussion following each focus group. Next, team members (EAB, AL, BRO, DK, MDG) performed content analysis by independently marking and categorizing key words, phrases, and texts to identify codes [35, 36]. The research team applied descriptive coding to describe the data and analytic or theoretical coding to explain why what was occurring in the data might be happening [37]. Transcripts were reviewed to resolve discrepancies through group consensus (EAB, AL, BRO, DK, MDG). No negative or deviant cases were excluded from the analysis [38]. This process continued until saturation was reached; that is, until no new codes emerged. After all transcripts were coded and reviewed, one member of the research team (EAB) entered the coded transcripts in NVivo 10 software (QSR International, Victoria, Australia) to further organize and group codes. The team (EAB, AL, BRO, DK, MDG) then met to agree on final themes, or patterns, that linked substantial portions of the data across focus groups [39]. The research team (EAB, AL, BRO, DK, MDG) selected themes that characterized the participants’ perceptions and experiences from data that occurred multiple times, both within and across transcripts. Each theme included words expressed by the participants to demonstrate that the concepts were grounded in the data. After the team coded and reviewed all transcripts, three clinician/researchers (TRF - family physician, EHG - clinical exercise physiologist, JW - clinical exercise physiologist) reviewed the codes and themes to achieve researcher corroboration [40].

### Rigor

To support credibility (validity), we triangulated the investigators with experts from different disciplines (i.e., health behaviorist/qualitative methodologist, health psychology, medicine, exercise physiology); analyst triangulation provided a check on selective perceptions as well as a means to identify blind spots in the analysis. Further, five participants reviewed the findings to achieve participant corroboration [41]. Transferability (external validity) was supported via rich descriptions and verbatim quotations included in the data. To support dependability (reliability) of the data, a researcher not involved with the study conducted an external audit to examine both the process of the research study and to evaluate whether or not the findings, interpretations and conclusions were supported by the data [38]. Confirmability (objectivity) of the data was supported via tracking the decision-making process with an audit trail, which is a detailed description of the research steps conducted from the development of the project to the presentation of findings [42].

## Results

Thirty-two former collegiate athletes (24.5 ± 2.9 years old, 59.4% female, 87.5% Non-Hispanic white, estimated response rate 9.1%; see Table 2) participated in 7 total focus groups (4 female focus groups and 3 male focus groups). Note, one of the male focus groups had 3 participants and another had 2 participants. These focus groups were supposed to have more male participants; however, several individuals did not attend leaving a small number of participants. We chose to move forward and conduct these focus groups with the small number of participants; the data collected were adequate and of high quality.

**Table 2:** Demographic and Collegiate Sport Characteristics of Study Participants (*n* = 32)

Variable	n (%)
Age (years)	24.5 ± 2.9
Education (years)	17.3 ± 1.1
Enrolled in Master’s degree program	15 (46.9)
Enrolled in Doctor of Philosophy program	3 (9.4)
Enrolled in Doctor of Osteopathic Medicine program	14 (43.8)
Gender	
Female (%)	19 (59.4)
Male (%)	13 (40.6)
Race (%)	
Non-Hispanic White	28 (87.5)
Asian	1 (3.1)
Black	1 (3.1)
Hispanic	1 (3.1)
Mixed Race	1 (3.1)
Competed in collegiate sport(s) (years)	3.6 ± 0.8
Division Level	
Division I (%)	14 (43.8)
Division II (%)	6 (18.8)
Division III (%)	9 (28.1)
Traveling Club Team (%)	3 (9.4)
Competed in collegiate sport with head gear (%)	12 (37.5)
Athletic Scholarship (%)	18 (56.3)
Training in concussion recognition with collegiate program (%)	12 (37.5)

On average, participants competed in collegiate sports for 3.6 ± 0.8 years. A little over half of participants (*n* = 18; 56.3%) received an athletic scholarship while competing in their sport. Participants competed in the following sports: soccer (*n* = 4), track and field (*n* = 4), football (*n* = 3), tennis (*n* = 3), basketball (*n* = 3), field hockey (n = 3), baseball (*n* = 2), wrestling (*n* = 2), lacrosse (*n* = 2), rugby (*n* = 2), softball (*n* = 2), volleyball (*n* = 2), swimming (*n* = 2), ice hockey (*n* = 1), and golf (*n* = 1), with four participants competing in multiple sports.

A history of 48 head injuries were self-reported, with several participants reporting multiple concussions. Fifteen participants (46.9%) reported a concussion accompanied by a loss of consciousness and/or confusion, and of these, only ten (32.1%) reported that their concussion was medically diagnosed. None of the participants reported having early or late seizures accompanied with their concussions; however, two participants reported a concussion requiring an overnight hospital stay.

### Focus group themes

Three predominant themes emerged from the focus group discussions: *Concussions are Part of the Game,* *Hiding Concussion Symptoms*, and *Misconceptions about Concussions in Low Contact Sports*. We included identifiers, indicating focus group number, gender, and ID, with quotations illustrating the themes.

#### Theme 1: Concussions are part of the game

Participants in all 7 focus groups recognized the frequency and seriousness of concussions. Interestingly, most participants believed that concussions were inherent in sports, particularly for collision sports and high contact sports like football, soccer, ice hockey, and rugby. They felt that by agreeing to play a sport, they were accepting the inherent risk of concussions, as evidenced by the following quotations:

“I think that certain sports [there] are inherent risks that you’re going to be hitting your head more, like hockey, football, soccer, and sports like that.” (Focus Group 2, Female ID 6)

“In general, I think at least like for my team in rugby, it’s the mentality of buck up, whatever it is, and concussions are going to happen so just deal with it.” (Focus Group 4, Female ID 16)

“I think everyone would kind of agree with this, by agreeing to play a sport and be a part of the team you're kind of taking in those risks …. It comes with playing the sport.” (Focus Group 5, Male ID 23)

“It almost seems like part of the game. It's like yeah, you're playing football. You get hit. It hurts. You've got to be tough.” (Focus Group 6, Male ID 30)

Nearly all participants expressed a desire to keep playing even if they witnessed or experienced a concussion themselves. Despite knowing the risks, participants cited passion and competitiveness as reasons for playing with a concussion. For example, one male participant and one female participant described their rationale for continuing to play if and when they had a concussion:

“I would probably play myself if I had a concussion and it was a big game. Yeah, I wouldn’t care. It’s more about the competition than being safe. Even though there are high risks and I know what the risks are, I’d probably still play through it, and that’s probably not a good idea.” (Focus Group 7, Male ID 31)

“I knew exactly what the risks were, especially when it happened my junior year because I was much farther into my education, but what I logicked [sic] out with myself was, I play tennis, there’s not a lot of things I could contact again, and I mean if I die, I die doing something I love, worst-case scenario.” (Focus Group 2, Female ID 9)

Importantly, some participants did not appear to fully understand what qualified as a concussion. Many referenced “seeing stars” or “black shiny stuff” at one point or another but continued to play through it. They rationalized this as head injuries were an expected part of playing sports:

“It’s [concussion] the sport. We don’t think of it as a concussion. I don’t want to say every time like I’ve seen stars from a head [hit] or that it happened hundreds of times in wrestling, but that to me isn’t a concussion. That’s just when you hit your head like at the house or something like that. I didn’t lose consciousness or anything like that, just kind of got bumped a little bit. I mean, if that is a concussion, then [expletive].” (Focus Group 6, Male ID 29)

#### Theme 2: Hiding concussion symptoms

Participants in each focus group discussed hiding concussion symptoms in order to avoid being taken out of or missing games. Some participants withheld information from coaches and trainers while others misrepresented or fibbed about their concussions. The following series of quotations demonstrates the frequency of hiding concussion symptoms in the former collegiate athletes in the study:

“I got to the point where, I mean I know it’s wrong, but you’re an athlete and you want to get back in there, so you flub some stuff.” (Focus Group 2, Female ID 9)

“I myself did this and other people; we just wouldn’t tell people if we were injured until after the game.” (Focus Group 4, Female ID 16)

“I know I did that myself sometimes because I wanted to play. That’s why you're there and you knew if you showed these symptoms that you’d get pulled, so you’d just sort of hide it or say nothing’s wrong when they come up and talk to you.” (Focus Group 5, Male ID 27)

“I’d say we’d all experienced it at one point or another. And I remember actually one guy specifically saying like, ‘Oh, did you see the black shiny stuff?’ I was like yeah. He was like, ‘Oh, okay.’ And that was that. We just kept playing, but I never told coaches or trainers or anyone. I guess I thought that if I did, then they were going to make me come out of the game and stop playing. And I felt like I needed to be there and I was still able to do it well enough to be in there. So I was like, oh man, I don’t want to come out because if I do, then I might have to miss the rest of the game. I was like well, I’ll just tough it out and see what happens. Hopefully, I’ll make it the rest of the game. I did so.” (Focus Group 6, Male ID 30)

Participants also acknowledged that hiding concussion symptoms was possible in part because most symptoms were indiscernible to the outside observer. As articulated by these two participants:

“It’s one of those injuries where there aren’t a lot of symptoms other than what’s reported. For example, an ankle, there’s bruising and swelling. You know, it’s hurt. But I think pressure from coaches, pressure from other players, and people just want to play, I think that’s a large part of [why] people play. Other people play through other injuries even when they're bad and they can get worse, but I think this is one of them that, because there’s no physical sign, there’s no bruising … you don’t even look bad.” (Focus Group 7, Male ID 31)

“You know it’s serious, but at the same time, I feel fine. It’s happened before, whatever. We’re all pretty intelligent, but we still have those innate thoughts of ‘Is it really that big of a deal?’ It’s hard to see the long, long road that I am going to lose memory quicker or something crazy like that. We can’t put it all together in one big piece … You probably should say something, but at the same time if you’re not having the symptoms of throwing up and vomiting, you’re not going to have the drive to really get pushed to say it [to a coach].” (Focus Group 6, Male ID 29)

Along these lines, several participants speculated that some athletes may hide concussion symptoms so as to not disappoint coaches and teammates. Participants explained that the invisibility of concussions called into question an athlete’s toughness and dedication to the team. For example, these participants described circumstances in which a player’s reputation may influence coaches’ and teammates’ impression of an athlete’s concussion:

“I think concussions are hard because a lot of what your teammates and coaches will think will probably reflect on what you’ve shown thus far in terms of how you respond to difficult situations or injuries. So if you’re thought of as that tough kind of person, they’re not going to doubt your concussion. But if maybe you’re looked at as being softer, they’re going be like, oh, this is just typical – this is what they do, they’re fine. Because a concussion isn’t something you can physically see, like a broken arm or something, so it’s something that people can doubt.” (Focus Group 3, Female ID 10)

“Someone who’s commonly hurt, you’d kind of like roll your eyes at them. But somebody who’s never hurt, I think that they would be treated differently.” (Focus Group 3, Female ID 17)

“Well, it depends on the person. [Laughter from the group]. If the person faked a lot of stuff, but if the person worked hard and was a pretty tough individual, you’d think all right, he must be hurting there.” (Focus Group 5, Male ID 20)

#### Theme 3: Misconceptions about concussions in low contact sports

While all participants were familiar with the incidence of concussions in sports, many did not understand that concussions could occur in any and all sports. Some participants felt that concussions were limited to only collision sports or high contact sports. Further, athletes in low contact sports or sports with minimal physical contact with other athletes (e.g., tennis, track and field, swimming) who experienced concussions, attributed their injuries to personal clumsiness rather than their sport. As demonstrated by these quotations:

“I threw a shot put and it didn’t go away, it went in my head because I didn’t know what I was doing, yeah. But I’m clumsy.” (Focus group 2, Female ID 7)

“People are like, ‘You play tennis and you had a concussion?’ It’s like, okay, I’m kind of clumsy, but yeah, it can happen … I mean what are the odds.” (Focus group 2, Female ID 9)

“I think it’s more about perspective, so you could look at it two ways. Football is more likely to get head injuries, so if someone had a concussion oh, okay, that makes sense. It was football. On the other hand, if it happens to someone in track, it’s like oh, wow, really? Okay. I guess it’s a fluke, but it’s probably not as bad as someone who would get it in football just because of the high contact or high speed, high force, et cetera.” (Focus group 7, Male ID 32)

Additionally, a few participants chose not report their head injuries to coaches and/or trainers because they felt it was embarrassing to have a concussion in a low contact sport. For example, this female participant described a head on collision with her teammate while swimming sprints in the same lane of the pool:

“I ran into someone head-on and we were both like sprinting and I had a headache. No one told me I had a concussion, I didn’t want to go to the trainer … but I was really confused. I had a really bad headache the whole week and I just had trouble thinking and stuff … That’s why it’s really embarrassing, because who does that?” (Focus group 3, Female ID 14)

## Discussion

In this qualitative focus group study, we explored former collegiate athletes’ understanding of concussions and their motivations behind concussion non-disclosure in sports. Our findings confirm a relatively high rate of concussions among former collegiate athletes, with a history of 48 total head injuries and 10 self-reported medically diagnosed concussions. Participants believed that concussions were part of sports, and that by agreeing to play a sport they were accepting the inherent risk of concussions. Of particular importance, many were not familiar with concussion symptoms and what constituted a concussion. These participants referenced symptoms of concussions (e.g., seeing stars) but continued to play through them. Participants also shared that they often hid concussion symptoms from coaches and trainers in order to avoid being taken out of or missing games. Participants explained that they were able to hide their concussions because most symptoms were invisible to coaches and trainers. Lastly, several participants did not understand that concussions could occur in all sports including low contact sports. The former athletes who participated in low contact sports and experienced concussions attributed their concussions to personal clumsiness rather than their sport. These findings highlight the importance of continued concussion awareness education among collegiate athletes, especially for athletes in low contact sports. Further, these findings draw attention to the importance of obtaining a detailed medical history so that family medical providers can integrate a history of concussions in current and future treatment plans.

Our research builds on earlier work exploring concussion non-disclosure among athletes. One study with high school athletes found that participants did not report concussions due to perceived lack of severity, not wanting to be removed from practice and/or a game, and not wanting to let down teammates and/or coaches [32]. Another study found that athletes did not report concussions because they lacked access to athletic trainers or other medical professionals, did not fully understanding the consequences of concussions, expressed a desire to play through the pain, and swore allegiance to the team [30]. Interestingly, a recent study found that US high school athletes with access to an athletic trainer had more knowledge about concussions; however, increased knowledge was not associated an increased likelihood to report suspected concussions [43]. A study with male collegiate football players’ identified minimization of risk, misconceptions about the long-term effects of concussions, diagnostic uncertainty, pressure from coaches, and athletic and social identity as reasons for not reporting concussions [31]. Finally, a study evaluating the effect of mandated US high school concussion education showed that while the number of athletes reporting that they received concussion education increased, the number of athletes reporting that they sought medical attention did not increase [44]. Although our study only included former collegiate athletes, almost all collegiate athletes also participate in high school athletics, so our findings within this studied can be applied to high-school athletics as well. These same themes emerged in our focus groups, which suggest that high school and collegiate athletes lack a complete understanding of concussions including concussions are not a normal part of playing a game, what constitutes a concussion, and the long-term consequences of a concussion or repeated concussions. Providing access to athletic trainers, which is standard practice in collegiate sports in the US, may improve concussion knowledge [43]. In addition, efforts to develop and disseminate evidence-based concussion education programs are needed to increase knowledge of symptoms and improve self-reporting of concussions.

Worldwide, millions of students and young adults participate in sports. Given the high rate of sports-related concussions, family medicine providers should ask adolescents and young adults about prior experiences with head injuries at sport physicals. This is particularly important during the preparticipation sports evaluation [45]. In athletes with a history of concussions, providers should determine the number of concussions, how they occurred, whether or not they lost consciousness, how long the symptoms lasted, and how soon they went back to playing the sport [45]. Providers should explain to patients that they are asking these questions because it is important to have a complete history of head injuries, and that they are not trying to keep them from playing sports. If a patient appears hesitant, providers should reiterate that they will not interfere with them playing sports, and that they only want to know their complete history in order to provider better care for them in the future. In addition, providers should perform a complete neurologic exam on athletes with a history of concussions [45]. If an athlete shows signs and symptoms of a concussion or postconcussion syndrome, the athlete should not be cleared for participation until his/her symptoms have resolved [45].

For adult patients, family medicine providers should ask if they engaged in high school or collegiate athletics. If yes, providers should ask if they were ever hit so hard they saw stars, could not remember the next play, or were asked to sit on the bench for a few minutes. Another approach is to ask patients to describe their hardest hit to the head. Follow-up questions should include if they received medical help or were taken to the hospital. This line of questioning is important given many of the participants in this study were not familiar with what constituted a concussion, and family practitioners need this information in order to provide longitudinal care for their patients. Further, symptoms of chronic traumatic encephalopathy (CTE) typically occur eight to ten years after repetitive head trauma [17, 46–49]. Thus, providers need to probe patients about a history of head injuries before they present with behavioral, mood, and/or cognitive symptoms of CTE. Along these lines, family medicine providers need to be familiar with current principles of concussion diagnosis and management to prevent long-term consequences like CTE. However, recent research shows gaps in the knowledge of concussion diagnosis and management among primary care providers [50–53]. Thus, continued efforts are needed to train and educate current and future providers to improve the care of athletes with head injuries.

### Limitations

Study limitations include the homogeneity of the study sample with regards to setting, sample size, race/ethnicity, and self-reported data. The study was conducted at one Midwestern university, with a small number of former collegiate athletes. Next, self-reported data were vulnerable to social desirability bias. To minimize bias, the researchers informed participants that their responses were confidential and could not be linked back to their personal identity. The researchers also emphasized the voluntary nature of participation and explicitly informed the participants that their responses had no bearing on their student status. Further, the study sample was predominantly white (87.5%); thus, future mixed method is needed to confirm the themes in a larger, more diverse athlete population. Lastly, the authors chose former collegiate athletes as participants in the study to enable them to speak openly about their experiences with concussions without fear of repercussion from coaches, trainers, or teammates. However, this choice may have resulted in different findings compared to a population of current athletes.

## Conclusions

Recent research on CTE underscores the need for family medicine providers to address head injuries across the lifespan. Family medicine providers are uniquely positioned to address the risks associated with concussions at all levels of sport participation – before, during, and after. Family medicine providers have the most frequent opportunities to speak with athletes of all ages at well-patient visits, sick visits, sport physicals, etc. This can include youth athletes in low and high contact sports as well as athletes in recreational or intramural leagues. In office discussions should include routine questions about hard hits to the head and “seeing stars,” not necessarily concussions given that many athletes have an incomplete understanding of concussions, and talk about the signs and symptoms of concussions. By not being affiliated with the team, family medicine practitioners can provide a non-biased third-party evaluation on concussion, perhaps improving concussion recognition and treatment in athletes across sports and ages. Future research is needed to create a simple questionnaire designed to measure patients’ “bumps, dings, seeing of stars” in family practice to supplement the more sophisticated concussion screening tools. In addition, qualitative research with current high school and collegiate athletes is needed to inform a line of questioning for family medicine providers to encourage current athletes to disclose head injuries to providers.

## Abbreviation

CTE: chronic traumatic encephalopathy
